# Citrus flavonoids repress the mRNA for stearoyl-CoA desaturase, a key enzyme in lipid synthesis and obesity control, in rat primary hepatocytes

**DOI:** 10.1186/1476-511X-10-36

**Published:** 2011-02-23

**Authors:** LaNita A Nichols, Daniel E Jackson, John A Manthey, Shivendra D Shukla, Lené J Holland

**Affiliations:** 1 Department of Medical Pharmacology and Physiology, University of Missouri School of Medicine, Columbia, Missouri 65212, USA; 2 United States Department of Agriculture, Agricultural Research Service, Citrus and Subtropical Products Laboratory, Winter Haven, Florida 33881, USA

## Abstract

Citrus flavonoids have been shown to decrease plasma lipid levels, improve glucose tolerance, and attenuate obesity. One possible mechanism underlying these physiological effects is reduction of hepatic levels of the mRNA for stearoyl-CoA desaturase-1 (SCD1), since repression of this enzyme reduces hyperlipidemia and adiposity. Here, we show that citrus flavonoids of two structural classes reduce SCD1 mRNA concentrations in a dose-dependent manner in rat primary hepatocytes. This is the first demonstration of repression of SCD1 by citrus flavonoids, either *in vivo *or in cultured cells. Furthermore, it is the first use of freshly-isolated hepatocytes from any animal to examine citrus flavonoid action at the mRNA level. This study demonstrates that regulation of SCD1 gene expression may play a role in control of obesity by citrus flavonoids and that rat primary hepatocytes are a physiologically-relevant model system for analyzing the molecular mechanisms of flavonoid action in the liver.

## Background

Understanding the molecular mechanisms that regulate lipid synthesis and deposition is of paramount importance, since obesity increases the risk of prevalent, life-threatening diseases such as diabetes and atherosclerosis. An intriguing model proposes that obesity is attenuated by lowering the amount of hepatic and/or adipose stearoyl-CoA desaturase-1 (SCD1), the rate-limiting enzyme in biosynthesis of monounsaturated fatty acids, which are preferred for triglyceride assembly [1]. This model is supported by gene knockout or knockdown studies, in which reduction of SCD1 mRNA levels restricted adiposity, insulin resistance, and hepatic lipid accumulation in rodents [2-5]. Conversely, elevated SCD1 levels in humans were associated with high plasma lipid concentrations, elevated hepatic lipid synthesis, obesity, or familial combined hyperlipidemia [6-9].

**Figure 1:**
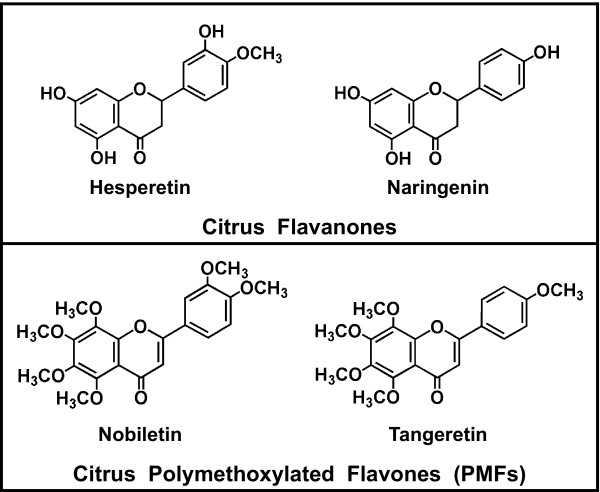
**Structures of two classes of citrus flavonoids**.

In the quest for therapies to alleviate obesity and associated illnesses, citrus flavonoids (Figure 1) are particularly promising, since a large body of research in humans and animals has shown hypolipidemic and/or antidiabetic effects of citrus fruits and juices [10-12], as well as purified flavonoids [12-20]. To examine the molecular mechanisms of citrus flavonoid action in more detail than is possible *in vivo*, the human hepatoma HepG2 cell line has been used extensively to establish that citrus flavonoids act through multiple pathways to reduce hepatic lipid secretion, and that the effects are consistent with physiological responses to these compounds in humans and animals [21-26]. Our previous work showed that citrus flavonoids regulated transcription of the low-density lipoprotein receptor (LDLR) gene in HepG2 cells, and that the DNA binding site for the transcription factor, sterol regulatory element binding protein (SREBP), was necessary for the regulation [27]. This work was the first direct demonstration that citrus flavonoids act at the level of hepatic gene transcription. Although the experimental manipulability of HepG2 cells has facilitated the analysis of underlying molecular mechanisms, it is desirable to use primary hepatocytes, since they more closely represent the physiology of intact liver. However, we are aware of only one published experiment in which citrus flavonoid action, specifically inhibition of apolipoprotein B secretion, was demonstrated in primary liver cells [21]. Therefore, the present study developed the use of isolated hepatocytes for examining hepatic effects of citrus flavonoids at the mRNA level. We chose to examine regulation of SCD1 mRNA because of the hypothesis that repression of SCD1 plays a key role in control of obesity and diabetes [1], and because of the recent report of citrus flavonoid attenuation of adiposity and insulin resistance in mice fed a high-fat diet [20].

## Methods

### Animals, primary hepatocyte isolation, and flavonoid treatment in culture

Male Sprague Dawley rats (Charles River Laboratories, Wilmington, MA) were used at 12-17 weeks of age, following protocols that conform with NIH guidelines and were approved by the University of Missouri Animal Care and Use Committee. Hepatocytes were isolated by collagenase perfusion [28] and cultured as described in Additional file 1-Detailed methods. Hesperetin (*≥* 95% pure) was from Sigma. Nobiletin was purified from tangerine peel and recrystallized twice to yield a purity of >99% [29]. Flavonoid stock solutions (50 mM) were prepared in dimethyl sulfoxide, the final concentration of which was 0.3% (v/v) in flavonoid-treated and control cells.

### RNA purification and analysis by molecular hybridization or quantitative real-time polymerase chain reaction (qRT-PCR)

RNA purification and molecular hybridization were conducted as described in Additional file 1-Detailed methods. Total RNA (20 μg/sample) was size fractionated on a formaldehyde gel and transferred to GeneScreen. Single-stranded cDNA probes for SCD1 and eukaryotic initiation factor 3H (EIF3H) mRNAs (Integrated DNA Technologies, Coralville, IA) (Table 1) were labeled, hybridized to the membrane, and detected by phosphorimaging. SCD1 mRNA was normalized to EIF3H mRNA, to correct for variable gel loading and any general flavonoid toxicity at higher flavonoid concentrations. The normalized results for treated samples are expressed as percent of the untreated control. qRT-PCR was carried out with SYBR-Green-based methodology (see Additional file 1-Detailed methods), using primer pairs for SCD1 or EIF3H (Table 1).

**Table 1:** Sequences of hybridization probes and qRT-PCR primers

Name	DNA Sequence
	**Hybridization Probes**
	5' 3'
**SCD1 (AS)^1^**	^1007 ^GTGGTGAAGTTGATGTGCCAGCGGTACTCACTG ^975^
**EIF3H (AS)^2^**	^1034 ^GGCAGTGAACTCCTTGATGTTCTGGCAGTAAGTGTT ^999^

	**qRT-PCR Primers**
	5' 3'
**SCD1 (S)^1^**	^26 ^GAAGCGAGCAACCGACAGCCAC ^47^
**SCD1 (AS)^1^**	^180 ^GTCTTCTTCCAGATAGAGGGGCAC ^157^
**EIF3H (S)^2^**	^850 ^AACACCAGTATCAGCAGCGTCG ^871^
**EIF3H (AS)^2^**	^1027 ^AACTCCTTGATGTTCTGGCAGTAAGTG ^1001^

^1 ^Sequence and numbering based on rat SCD1 (GenBank ID: NM_139192.2)

^2 ^Sequence and numbering based on rat EIF3H (GenBank ID: NM_198751.1)

SCD1 and EIF3H hybridization probes are located within the protein-coding regions. The PCR-amplified sequence from EIF3H mRNA includes most of the 33-mer used as the EIF3H hybridization probe. The PCR-amplified sequence from SCD1 mRNA does not overlap with the SCD1 hybridization probe, because of the necessity to avoid potential cross reactivity with SCD2 mRNA, but it does produce an amplicon that is mostly within the protein-coding region. The SCD1 primer set does not match the SCD2 mRNA sequence (GenBank ID: NM_031841.1), and cloning and sequencing of the product generated by qRT-PCR confirmed that the amplified sequence was SCD1.

## Results

### Verification of hybridization probes for SCD1 and EIF3H mRNAs

**Figure 2:**
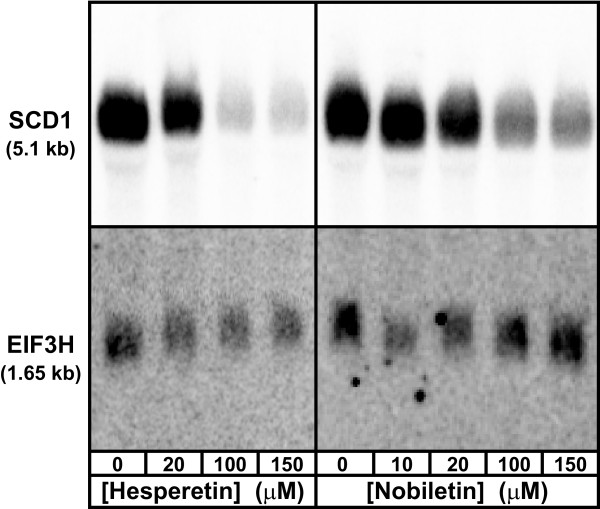
**Specificity of hybridization probes for SCD1 or EIF3H mRNA in rat hepatocyte RNA**. Rat hepatocytes were treated with vehicle, 20-150 μM hesperetin, or 10-150 μM nobiletin for 20 h. Total RNA was hybridized with cDNA probes for SCD1 mRNA or the normalizer, EIF3H mRNA. Apparent sizes of the RNAs are denoted on the left in kilobases (kb).

Rats have two SCD genes, SCD1 and SCD2 (sometimes called SCD). Hybridization of size-fractionated rat hepatocyte RNA with the SCD1 probe yielded a single RNA band of ~5,100 bases (Figure 2), similar to the previously-described ~5,900 bases [30]. These sizes are larger than the reported 4475 bases (GenBank ID: NM_139192.2), but that sequence is not necessarily full length. Although our hybridization probe matches SCD2 mRNA (GenBank ID: NM_031841.1), it is unlikely that the detected RNA is SCD2, since that isoform was completely undetectable in rat liver tissue [30]. qRT-PCR experiments below confirmed that the SCD isoform expressed in rat hepatocytes was SCD1. For normalization we used mRNA for the housekeeping protein, EIF3H. The EIF3H probe hybridized with a single RNA species of ~1,650 bases (Figure 2), which is compatible with the reported 1,243 bases (Genbank ID#: NM_198751.1).

### Dose-dependent repression of SCD1 mRNA levels by hesperetin or nobiletin in rat hepatocytes

**Figure 3:**
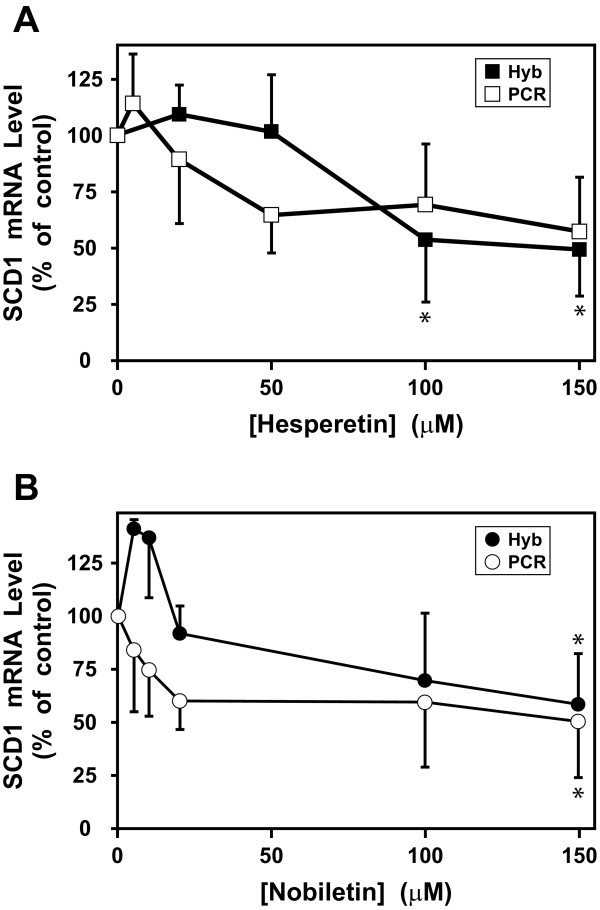
**Dose-dependent repression of SCD1 mRNA levels in rat hepatocytes by hesperetin or nobiletin**. Rat hepatocytes were treated with vehicle or 5-150 μM hesperetin or nobiletin for 18-20 h in four independent experiments. mRNAs were quantitated by hybridization (closed symbols) or qRT-PCR (open symbols). Effects of increasing doses of (A) hesperetin or (B) nobiletin on normalized SCD1 mRNA levels are expressed as percent relative to the untreated control. At each flavonoid concentration, n = 3 or 4, and the error bars represent SD. Each experimental condition was compared back to the control by one-way ANOVA with Dunnett's post test using InStat (GraphPad, La Jolla, CA). An asterisk indicates a statistically-significant difference from the untreated control (*P *≤ 0.05).

To represent the flavanone class, we used hesperetin (Figure 1), since it was more effective than naringenin in HepG2 cells [22]. For the polymethoxylated flavone class, which has been shown to be more potent (i.e. effective at lower doses) than flavanones *in vivo *[19] and in HepG2 cells [23,27], we chose nobiletin, since it was more effective than tangeretin in HepG2 cells (our unpublished data). For quantitative analysis, mRNA concentrations were assayed both by hybridization, which allowed assessment of RNA integrity and correct size (as in Figure 2), and by qRT-PCR, which allowed more rapid quantitation and exclusive detection of the SCD1 isoform. For 150 μM hesperetin, repression of SCD1 mRNA reached 49% (by hybridization) or 57% (by qRT-PCR) compared to the untreated control (Figure 3A). The inhibition was statistically significant (*P *≤ 0.05) at 100 and 150 μM hesperetin by the hybridization assay. The qRT-PCR data did not quite reach statistical significance, but the results were very similar to those in the hybridization assay. For 150 μM nobiletin, the inhibitory effect was 58% (by hybridization) or 50% (by qRT-PCR), which was statistically significant (*P *≤ 0.05) by both assays (Figure 3B). At low concentrations of nobiletin (5-10 μM), there is some difference in the pattern of the response by the two assays, but none of the effects in this concentration range were significantly different from the control. Despite the differences at low doses, the overall trend is a decrease in SCD1 mRNA with increasing concentrations of nobiletin, similar to that of hesperetin.

## Discussion

The citrus flavonoid repression of SCD1 mRNA levels described here is compatible with the recent report that naringenin reduced adiposity and weight gain in mice after 4 weeks [20], based on the model that SCD1 plays an important role in obesity control [1]. The *in vivo *effects of flavonoids were proposed to be due to a reduction in the amount of SREBP1 [20]. However, previous work in HepG2 cells indicated that citrus flavonoids stimulate, rather than repress, SREBP levels after short term treatments [21,27]. This apparent discrepancy may be explained by well-established mechanisms whereby SREBPs stimulate many genes that elevate lipids and cholesterol production [31]. Cholesterol then sequesters SREBPs in an inactive form, which leads, in the long term, to decreased expression of genes that were initially induced, including the SREBP genes themselves [31-33]. Thus, SREBP effects on hepatic lipid handling *in vivo *are a complex balance between opposing actions and feedback mechanisms [31].

Because citrus flavonoids elevate SREBPs in HepG2 cells, the simplest prediction is that these compounds stimulate SREBP activity in primary rat hepatocytes. However, our observation of the repression of SCD1 mRNA is not compatible with this prediction, since the SCD1 gene is a positive target for both SREBP1 and SREBP2 [32,33]. Thus, our results suggest that, in rat liver cells, either the flavonoids reduce SREBPs or repression of SCD1 mRNA occurs by SREBP-independent mechanisms. A study with a different flavonoid, the soy isoflavone genistein, also showed repression of SCD1 mRNA levels in HepG2 cells [34]. This repression correlated with a 50% decrease in nuclear SREBP1 and a 5-fold increase in nuclear SREBP2, but these conclusions are not definite since the particular antibody used should not recognize the mature N-terminal portion of SREBP2 in the nucleus, and data from multiple experiments were not reported [34]. Another group found that soy isoflavones increased the amount of the C-terminal mature portion of SREBP2 in whole cell extracts of HepG2 cells after 24 h, but SREBP1 levels did not change [35]. Because of this variability regarding flavonoid effects on SREBP levels in HepG2 cells, the rat primary hepatocytes will be invaluable for deciphering the mechanisms underlying the complexities of regulation of both isoforms of SREBP, as well as the role of SREBP in flavonoid repression of the SCD1 gene.

Freshly-isolated hepatocytes allow a more thorough mechanistic analysis of flavonoid action than is possible *in vivo *and are more physiologically-relevant than tumor-derived HepG2 cells. A detailed molecular understanding is essential for evaluating the potency and efficacy of flavonoids of different structural classes and metabolic forms, so that ultimately the most effective flavonoid-based treatments can be used for combating atherosclerosis, diabetes, and obesity.

## Abbreviations

EIF3H: eukaryotic initiation factor 3H; LDLR: low-density lipoprotein receptor; qRT-PCR: quantitative real-time polymerase chain reaction; SCD: stearoyl-CoA desaturase; SREBP: sterol regulatory element binding protein.

## Competing interests

JAM receives a small portion of the annual payment to the United States Department of Agriculture for licensing of U.S. patents 6,184,246 and 6,987,125, which deal with the cardiovascular and inflammation protection actions of citrus polymethoxylated flavones. The other authors declare that they have no competing interests.

## Authors' contributions

LAN participated in experimental design and carried out experiments. DEJ carried out experiments. JAM supplied research expertise and carried out flavonoid purification. SDS supplied research expertise and experimental materials. LJH conceived of the study, participated in experimental design, carried out experiments, and drafted the manuscript. All authors edited the draft manuscript, and read and approved the final manuscript.

## Authors' information

*Department of Medical Pharmacology and Physiology, MA 415 Medical Sciences Building, University of Missouri School of Medicine, One Hospital Drive, Columbia, Missouri 65212, USA. Telephone: 1-573-882-5373. FAX: 1-573-884-4276.

## Supplementary Material

Additional file 1 **Detailed methods**. Methodological details for hepatocyte isolation and culture, RNA purification, molecular hybridization, and qRT-PCR. Click here for file
