# Supplementary material for: Citrus flavonoids repress the mRNA for stearoyl-CoA desaturase, a key enzyme in lipid synthesis and obesity control, in rat primary hepatocytes
Source: Lipids Health Dis. 2011 Feb 23;10:36. doi: 10.1186/1476-511X-10-36 (PMC3056818; doi:10.1186/1476-511X-10-36)
Supplement: Additional file 1 — Detailed methods. Methodological details for hepatocyte isolation and culture, RNA purification, molecular hybridization, and qRT-PCR. [file 1476-511X-10-36-S1.PDF]

Additional file 1-Detailed methods

Primary hepatocyte isolation and maintenance in culture

Rats were anesthesized with intraperitoneal injection of 8.3 mg ketamine and 1.7 mg xylazine per 100 g body weight. Hepatocytes were isolated by the collagenase-perfusion method [1], with the following modifications. The portal vein was cannulated and the liver perfused with Krebs-Ringers-bicarbonate (KRB) buffer (110 mM NaCl, 25 mM $NaHCO_3$, 12 mM KCl, 1.2 mM KH_2PO_4, 10 mM Hepes pH 7.4, 5 mM glucose) containing 0.25 mM EGTA. The inferior vena cava was cut below a loose suture to allow drainage of blood from the liver. The inferior vena cava was then ligated, the superior vena cava cannulated, and the liver perfused with recirculating KRB containing 3.0 mM $CaCl_2$, 0.5 mM $MgSO_4$, and Type 1 collagenase (Worthington, Lakewood NJ) at ~17,000 units/150 ml. The hepatocytes were washed in KRB containing 3.0 mM $CaCl_2$, 1.3 mM $MgSO_4$, and 0.5% bovine serum albumin (#A7906, Sigma, St. Louis, MO). Cell viability was 89% ± 4% (SEM). For collagen-coating of dishes, 10 ml of 40 μg/ml Type 1 rat tail collagen (BD Biosciences, San Jose, CA) in 0.02 N acetic acid was applied per 100 mm dish and incubated at 37°C under 5% CO_2 for 3 h. The plates were rinsed with PBS, the 37°C incubation continued overnight, residual liquid removed, and plates stored at 4°C. Liver cells were plated at 7×10^6 cells/10 ml/100 mm dish and maintained at 37°C under 5% CO_2 in Dulbecco's Modified Eagle's Medium containing 25 mM glucose (GIBCO, Carlsbad, CA or Hyclone, Logan, UT) and supplemented with 2 mM glutamine, 10% fetal bovine serum (Hyclone or Atlanta Biologicals, Lawrenceville, GA), and 100 units/ml penicillin plus 100 μg/ml streptomycin sulfate (GIBCO). Approximately 3-8 h after initial plating, the medium was replaced with fresh medium of the same composition plus flavonoids.

RNA purification and analysis by molecular hybridization

RNA purification and molecular hybridization were conducted as described in the Online Supporting Material in reference 2. Briefly, cells (without washing) were lysed in a total of 4 ml lysis buffer (Qiagen, Hilden, Germany) per 3 culture dishes for each flavonoid treatment. RNA was purified with the RNeasy system (Qiagen) followed by phenol extraction. Final yield of RNA ranged from 30-60 μg/100 mm dish and did not vary due to flavonoid treatments.

Quantitative real-time polymerase chain reaction (qRT-PCR)

qRT-PCR was carried out with SYBR-Green-based methodology as described in the Online Supporting Material in reference 2, with the following modifications. Superscript II or III (Invitrogen, Carlsbad, CA) was used for initial cDNA synthesis from rat hepatocyte total RNA. The qRT-PCR utilized cDNA template from 10 ng of RNA for the SCD1 reactions and 200 ng RNA for the EIF3H reactions. Optimized primer concentrations were 0.2 μM sense primer and 0.2 μM antisense primer for both SCD1 and EIF3H. The annealing/extension temperatures were 58ºC for SCD1 and 57ºC for EIF3H. Specificity of the primers was confirmed by melt curve analysis and presence of a single band of the correct size by gel electrophoresis. For quantitation, PCR efficiencies were determined from the cDNA synthesized from untreated samples in four separate experiments. The mean efficiencies were 97.0% ± 1.7% (SEM) for SCD1 and 93.2% ± 2.1% (SEM) for EIF3H.

References

1. Weng Y-I, Shukla SD: **Ethanol alters angiotensin II stimulated mitogen activated protein kinase in hepatocytes: agonist selectivity and ethanol metabolic independence.** *Eur J Pharmacol* 2000, **398**:323-331.
2. Morin B, Nichols LA, Zalasky KM, Davis JW, Manthey JA, Holland LJ: **The citrus flavonoids hesperetin and nobiletin differentially regulate low density lipoprotein receptor gene transcription in HepG2 liver cells.** *J Nutr* 2008, **138**:1274-1281.
